# Adult neurobehavioral outcome of hyperbilirubinemia in full term neonates—a 30 year prospective follow-up study

**DOI:** 10.7717/peerj.294

**Published:** 2014-03-04

**Authors:** Laura Hokkanen, Jyrki Launes, Katarina Michelsson

**Affiliations:** Institute of Behavioural Sciences, University of Helsinki, Helsinki, Finland

**Keywords:** Hyperbilirubinemia, Outcome, Neurobehavioral, Neonatal, Adulthood, Intelligence, Cohort, Prospective

## Abstract

**Background**. Neonatal hyperbilirubinemia (HB) may cause severe neurological damage, but serious consequences are effectively controlled by phototherapy and blood exchange transfusion. HB is still a serious health problem in economically compromised parts of the world. The long term outcome has been regarded favorable based on epidemiological data, but has not been confirmed in prospective follow-up studies extending to adulthood.

**Methods**. We studied the long term consequences of HB in a prospective birth cohort of 128 HB cases and 82 controls. The cases are part of a neonatal at-risk cohort (*n* = 1196) that has been followed up to 30 years of age. HB cases were newborns ≥ 2500 g birth weight and ≥ 37 weeks of gestation who had bilirubin concentrations > 340 µmol/l or required blood exchange transfusion. Subjects with HB were divided into subgroups based on the presence (affected HB) or absence (unaffected HB) of diagnosed neurobehavioral disorders in childhood, and compared with healthy controls. Subjects were seen at discharge, 5, 9 and 16 years of life and parent’s and teacher’s assessments were recorded. At 30 years they filled a questionnaire about academic and occupational achievement, life satisfaction, somatic and psychiatric symptoms including a ADHD self-rating score. Cognitive functioning was tested using ITPA, WISC, and reading and writing tests at 9 years of life.

**Results.** Compared to controls, the odds for a child with HB having neurobehavioral symptoms at 9 years was elevated (*OR* = 4.68). Forty-five per cent of the HB group were affected by cognitive abnormalities in childhood and continued to experience problems in adulthood. This was apparent in academic achievement (*p* < 0.0001) and the ability to complete secondary (*p* < 0.0001) and tertiary (*p* < 0.004) education. Also, the subgroup of affected HB reported persisting cognitive complaints e.g., problems with reading, writing and mathematics. Childhood symptoms of hyperactivity/impulsivity (*p* < 0.0001) and inattention (*p* < 0.02) were more common in HB groups, but in adulthood the symptoms were equal. The affected HB had lower scores in parameters reflecting life satisfaction, less controlled drinking, but not increased substance abuse.

**Discussion**. Our results indicate that neonatal HB has negative consequences in adult age. A prospectively collected cohort with strict inclusion criteria enables to control most of the bias factors involved with retrospective data. The control and HB groups were remarkably similar at birth in terms of medical data, and the growth environment of the children, as well as the parents’ social groups, education, size of family, type of housing at birth and at 9 years of age. Our findings bear resemblance to disorders of the fronto-striatal network, and also symptoms of the ADHD spectrum were frequent in the HB group suggesting a link of HB to other neurodevelopmental disorders.

## Introduction

Neonatal hyperbilirubinemia (HB) is a well-recognized risk for severe neurological disability and death (Ip et al., 2004). While it is at present reasonably well controlled in the developed countries, it is still a concern in economically compromised areas (Sgro, Campbell & Shah, 2006; Kaplan, Bromiker & Hammerman, 2011). The pathogenesis of encephalopathy by unconjugated bilirubin is poorly understood but neuronal damage may be linked to oxidative stress and a disruption of the blood–brain barrier (Palmela et al., 2011). Kernicterus refers to the severe neurodevelopmental consequences, but more subtle forms of bilirubin encephalopathy also exist (Shapiro, 2003; Johnson & Bhutani, 2011). The type and severity of the effects of HB have mostly been studied in relatively short follow-up studies ranging up to school age, where mild to moderate neonatal HB has appeared to be relatively, if not totally, benign (Culley et al., 1970; Newman & Klebanoff, 2002; Ip et al., 2004; Gamaleldin et al., 2011). There is still controversy however on the risks for neurodevelopmental disorders in later childhood (Shapiro, 2003; Kuzniewicz & Newman, 2009; Johnson & Bhutani, 2011; Koziol, Budding & Chidekel, 2013). To date, there are very few studies on neonatal hyperbilirubinemia followed up to early adulthood, and none beyond 20 years. The existing ones are based on retrospective analysis of birth data during military drafting of conscripts (Nilsen et al., 1984; Seidman et al., 1991; Ebbesen et al., 2010).

In a Norwegian study reporting results of 39 male conscripts at 18 years of age, the mean intelligence scores were comparable to the total conscript cohort, but seven subjects with a more severe hyperbilirubinemia had significantly lower scores than the national average (Nilsen et al., 1984). A study from Israel with a much larger sample (*n* = 1948) found no linear association between neonatal bilirubin levels and intelligence test scores or school achievement at 17 years of age, but the risk for low intelligence test scores was higher among full term males (but not females) with serum bilirubin levels above 342 µmol/l (Seidman et al., 1991). The most recent study was from Denmark, where no association between higher bilirubin levels and the risk of obtaining a neuropsychiatric diagnosis, or scoring lower in the cognitive test used in the drafting was seen in males with a median age of 18.8 years (Ebbesen et al., 2010). There was however a larger proportion of men found unfit for military service in the HB group. Also, only 25 subjects (6% of all hyperbilirubin cases) in the Danish study were exposed to bilirubin levels above 342 µmol/l, the cut-off value that has previously been regarded significant.

These studies have mainly relied on IQ data and only offer limited outcome information. Intelligence scores are often insensitive to executive dysfunction, learning deficits and affective disorders (Johnson & Bhutani, 2011). An association between neonatal jaundice and neurodevelopmental syndromes, e.g., ADHD has been suggested (Jangaard et al., 2008) but not proven (Kuzniewicz, Escobar & Newman, 2009). Also a link to autism and other disorders of psychological development has been presented in HB (Jangaard et al., 2008; Maimburg et al., 2010). In a national cohort from Denmark, full-term children exposed to jaundice as neonates had 56% to 88% greater risks of psychological development disorders, compared with children not exposed to jaundice (Maimburg et al., 2010). The conclusion of HB being of benign nature based on intelligence test findings may therefore be inaccurate. Very little is known about the vocational and social outcome of hyperbilirubinemia in adults.

We report here the 30 year results of a prospective follow-up study on a birth cohort of full term and normal weight newborns with hyperbilirubinemia (HB > 340 µmol/l) as the only birth risk factor whose adult outcome is compared to healthy, typically developed controls similarly followed-up. Our aim was to study if the cognitive and behavioral problems discovered in childhood continue in adulthood, and if they affect the educational, occupational, and social functioning as well as health and wellbeing of the adult subjects.

## Material & Methods

### Participants

The birth risk cohort originates from a single maternity hospital in Helsinki, Finland. During the recruitment period 1971–74, there were 22,359 consecutive births in this hospital, out of which 1196 (5.4%) neonates had at least one predefined risk: APGAR score lower than 7 at 5 or 15 min (*n* = 372), birth weight under 2000 grams (*n* = 317), jaundice with bilirubin > 340 µmol/l (*n* = 368), severe respiratory difficulties necessitating external ventilation (*n* = 161), neurological symptoms (*n* = 195), maternal diabetes (*n* = 95), infant hypoglycemia (*n* = 104), and severe infection (*n* = 36); 19% had more than one risk factor (Michelsson et al., 1978).

For the present study only full term neonates with hyperbilirubinemia were included. For inclusion, at least two serum bilirubin values of 340 µmol/l (20 mg/100 ml) or more were required. Children who had received blood exchange transfusion because of rapidly increasing serum bilirubin values were also included. Those who died during early childhood and those with severe disabilities (incl. kernicterus) were excluded from follow up (Michelsson, Donner & Lindahl, 1988). Additional exclusion criteria for the present study included gestational age below 37 weeks, or birth weight below 2500 g. There were 238 HB cases fulfilling these inclusion and exclusion criteria. All subjects were Caucasian and the families spoke Finnish.

The control subjects, also prospectively studied from childhood, were born in the same hospital during the study period, attended the same schools, and were free of any perinatal risk factors. For the present study those with gestational age at or above 37 weeks, birth weight at or above 2500 g and full assessment information from 9 years of age are included (*n* = 145).

Ethical review has been conducted over the course of the longitudinal study, and the latest approval was obtained from the Ethical Review Board of the Helsinki and Uusimaa hospital district in May 2013 (number 147/13/3/00/2013). All participants gave their written consent to the study.

### Methods and procedures

The family’s social and economic status, maternal risk factors, genetic traits, medical data about delivery and delivery complications was recorded prospectively. The socio-economic status was defined as the father’s occupational level (1 to 5, 1 being the highest category). Psychosocial distress score was formed to include poor housing conditions, divorces, relocations, alcohol abuse, unemployment, family conflicts, domestic violence, imprisonment, severe diseases or mental problems in family. Body-mass index was calculated as an indicator of the general health and development of the child.

The cognitive and behavioral problems in childhood were assessed when the children were 5 and 9 years of age. This coincides with periods before and after entering school (at the age of 7 in Finland). The assessment methods have been described in detail elsewhere (Hokkanen, Launes & Michelsson, 2013). To summarize, a structured neurological assessment was carried at 5 and at 9 years of age out using the Neurodevelopmental Screen developed by Michelsson et al. (Michelsson, Ylinen & Donner, 1981; Lindahl, Michelsson & Donner, 1988). The method is a modification of the test of Bax & Whitmore (1973) that examines visual acuity, squint, gross motor performance, fine motor performance, co-ordination and balance, involuntary movements when standing, upper motor neuron disturbances, articulation, language skills, perception, concentration and behavior. Other standardized tests and measures used at 5 and/or 9 years of age include the Test of Motor Impairment (Stott, Moyes & Henderson, 1972), the Dubowitz Intellectual development screening test (Dubowitz, Leibowitz & Goldberg, 1977) measuring perceptual and numerical skills, The Illinois Test of Psycholinguistic Abilities (Kirk, McCarthy & Kirk, 1968), The Draw-a-person test (Goodenough, 1926), the Frostig Developmental Test of Visual Perception (Frostig, Lefever & Whittlesey, 1966), Reading and spelling tests for Finnish children (Ruoppila, Roman & Vasti, 1968; Ruoppila, Roman & Vasti, 1969) and subtests from the Wechsler Intelligence Scale for Children (WISC, Wechsler, 1949).

A questionnaire was filled out by parents both at ages 5 and 9 regarding the parents’ work situation, existing developmental problems or disabilities within the family, the child’s home environment, development and skill acquisition, illnesses, school achievement and problem behaviors. The existing problems within the family that were probed were: delayed speech, delayed motor skills, clumsiness, reading and spelling difficulties, other school problems, inattention or short temperedness, squint, impaired hearing in childhood, mental retardation, mental illnesses and seizures, present or absent in other family members. In addition, the questionnaire at 5 years included 28 questions regarding the activity level, motor skills, communication and emotional affectivity of the child rated in a three point scale. At 9 years of age a questionnaire was also sent to the teachers concerning school achievement and the need of remedial teaching or a special class. Additionally, the teacher was asked to perform an evaluation of the pupil’s behavior and personality in class using 24 adjective pairs (e.g., observant—day dreaming) with a five point rating between the extremes, as well as five open-ended questions for additional information.

Based on the comprehensive assessments with measures described above, the children were classified into either having or not having different developmental disorders. The decisions were agreed upon at a clinical case conference between a pediatrician, child neurologist, a psychologist, and a speech therapist, who had performed the assessments. The results of these 5 and 9 year assessments have been published previously (Lindahl & Michelsson, 1986; Lindahl & Michelsson, 1987; Lindahl, Michelsson & Donner, 1988; Michelsson, Ylinen & Donner, 1981; Michelsson, Donner & Lindahl, 1988; Michelsson & Lindahl, 1984; Michelsson & Lindahl, 1987; Michelsson et al., 1988; Michelsson & Lindahl, 1993; Michelsson & Noronen, 1983). The diagnostic classification in the HB group were as follows: 21% had minimal brain dysfunction (MBD)—a diagnosis in use at the beginning of the cohort study defined as three or more concomitant deficits—30% had dyslexia, 27% perceptual disabilities (auditory, visual or undefined), 27% had motor impairment, 15% hyperactivity and attention deficit, 11% significant psychiatric and psychosomatic symptoms, 9% had neurological signs, and 5% had speech and language abnormalities (not including dyslalia only). For the purposes of the present study, a combined score of all the developmental findings was constructed and those with a score of 1 or above were considered to be affected with at least one of the diagnoses. To analyze the persistence of symptoms discovered in childhood, the subjects were divided into two groups (affected and unaffected), based on this classification.

Long term outcome at the age of 30 years was assessed with an extensive questionnaire prepared for the purposes of this study. Educational outcome was measured by questions regarding school achievement (graduation from first-level obligatory school, the need for remedial teaching, the final school grades earned, and the highest degree completed in secondary or tertiary education). Occupational achievement was measured with a question about the type of present employment (full-time, part-time, student, maternity leave, unemployed). Social functioning was assessed with a combined score measuring social satisfaction (three items) and social contacts (two items), higher value indicating more dissatisfaction. Life satisfaction, satisfaction with social relationships and satisfaction with social support received were measured with a 5 point scale (from very satisfied to very unsatisfied) and the social contact items included reported difficulty in making friends or maintaining social relationships (yes/no). General health was measured with a combined score of subjective health (rated with a five-point scale from very good to very poor), the number of doctors’ appointments (four-point scale), the use of medication for physical illnesses, and the presence of headaches or gastro-intestinal complaints (yes/no). The incidence of past traumas, fractures and concussions was also noted. Substance use (illicit drugs, alcohol consumption frequency, alcohol related problems) were also included in the questionnaire as indicators of health and well-being. In addition to categorical variables, an alcohol consumption score was constructed by combining items of regularly drinking more than once a month (dichotomized from a six-point scale from never to daily), excessive use expressed by family members (yes/no), excessive use estimated by self (yes/no) and reported incidences of driving while intoxicated (yes/no). The psychiatric status was measured with a combined score of reported psychiatric problems (presence or absence of depression, anxiety disorder, panic disorder, obsessive-compulsive traits, manic-depression, or sleep disorders), and the use of prescribed sedatives or hypnotics. The behavioural outcome was measured with the ADHD Current Symptoms Scale as well as the ADHD Childhood Symptoms Scale where each of the 18 DSM-IV (American Psychiatric Association, 1994) diagnostic symptom criteria is scored from 0 to 3 depending on the severity of symptoms (Barkley & Murphy, 1998). Cognitive outcome was measured with ongoing cognitive complaints (presence or absence of persisting subjective learning difficulties, writing difficulties, reading difficulties, perceptual problems, mathematical problems, speech problems, motor/dexterity problems).

**Figure 1:**
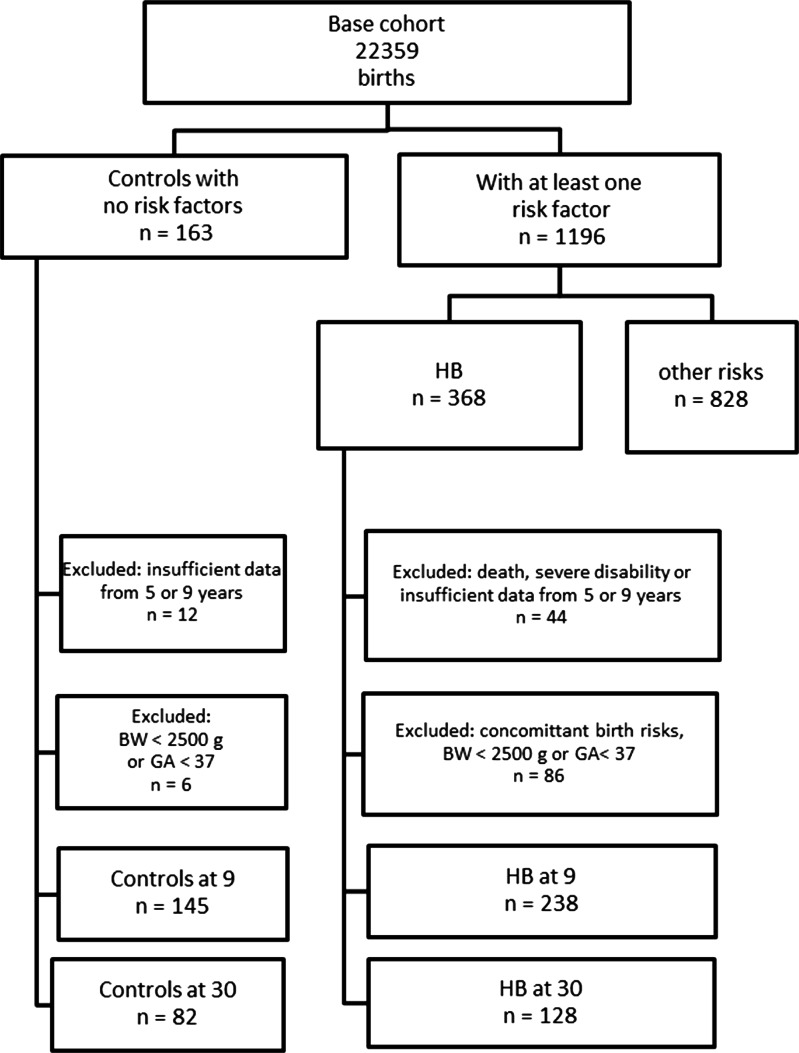
Flow chart of the birth risk cohort subjects and controls.

Of the cases fulfilling the criteria of the present study, 128 HB cases (54%) and 82 controls (57%) returned an adequately completed questionnaire at the age of 30 years. See Fig. 1 for the complete flow chart. We analyzed the possible attrition bias caused by the 54% compliance in the HB group by comparing the responders and the non-responders (data not shown). At birth, there was no difference between the groups in the parity, the percentage of small-for-date, apgar points, gestational weeks, birth weight, head circumference, gender ratio, mother’s smoking habits during pregnancy, or the family socio-economic status. The developmental classification score at the age of 9 years was similar in both groups.

### Statistical analysis

Statistical analysis was performed using the STATISTICA analysis software (StatSoft, Inc., 2013). For continuous outcome variables, a multiple analysis of variance (MANOVA) was used in three separate models that included variables of for birth data, cognitive tests at 9 years of age, and ADHD symptom scores at 30 years. Missing data was replaced by mean values of relevant groups. Differences between three groups were further tested by univariate analysis of variance (ANOVA) and pair-wise comparisons. Due to multiple comparisons, we used the Bonferroni method post-hoc test because of the conservative estimates it produces. The Kruskall–Wallis nonparametric analysis of variance was used for ordinal variables or when the distribution was skewed. For testing significance of two independent variables, the Mann–Whitney-U or the Kolmogorov–Smirnov two sample tests were used. Frequencies were calculated from contingency tables and Fisher’s exact test was used to test the significance in 2 × 2 tables.

## Results and Discussion

There were 57 HB cases with at least one neurobehavioral disability used for classification at 9 years of life (the affected-HB group) and 71 with no disabilities (the unaffected-HB group). The controls were similarly classified into unaffected (*n* = 70) and affected (*n* = 12) groups. The odds ratio was calculated as *OR* = 4.68 (95% confidence interval 2.21–10.11) for a subject with HB of belonging in the affected group as compared to controls.

The result indicates that the odds for a subject with neonatal HB being in the affected group was nearly five-fold compared to the controls. As ADHD, dyslexia and other developmental disabilities are each found in about 5%–10% of the general population (Shaywitz & Shaywitz, 2005; Polanczyk et al., 2007; Willcutt et al., 2010), finding individuals affected with, e.g., reading or writing impairment, motor difficulties, and attentional problems in our control group as well was expected. HB however greatly increases this risk.

To study if the neurobehavioral difficulties of childhood improve or disappear in adulthood, we continued to examine the affected and unaffected subgroup separately. The subgroups were based on categories that had been formed prospectively, independently from the present study. Because the aim was to compare the HB group with typically developed healthy subjects, controls affected with developmental disabilities were excluded from further analyses. The rationale was that a control group containing both affected and unaffected individuals would have created a bias in favor of unaffected HB and in disfavor of affected-HB cases, obscuring the potential differences.

### The management of neonatal hyperbilirubinemia

Table 1 shows the perinatal background data in the three groups. A statistically significant difference was found in MANOVA for the continuous variables (Wilks’ Lambda *p* < 0.0001) but in univariate ANOVAs only the 5 min apgar differed between the groups, with the control group actually being the lowest. There was a slight but statistically not significant over representation of girls in the control group (57%) and boys in the affected (61%) and unaffected (55%) HB group.

**Table 1:** Medical and birth data for the affected and unaffected groups with hyperbilirubinemia (HB) and for the healthy controls. The significances for differences between the three groups in univariate comparisons are also given.

	Affected-HB *n* = 57	Unaffected-HB *n* = 71	Controls *n* = 70	
	Mean (95% CI)	Mean (95% CI)	Mean (95% CI)	Univariate test across the three groups
Gestational age (weeks)	39.5 (39.1–39.8)	39.3 (38.9–39.6)	39.8 (39.5–40.2)	ns
Birth weight (g)	3505 (3376–3633)	3469 (3354–3585)	3538 (3428–3649)	ns
Head circumference (cm)	34.6 (34.2–34.9)	35.0 (34.7–35.3)	35.0 (34.7–35.4)	ns
Apgar 1 min	8.6 (8.4–8.8)	8.5 (8.3–8.8)	8.9 (8.7–9.2)	ns
Apgar 5 min	9.8 (9.7–10.0)	9.9 (9.7–10.0)	9.6 (9.4–9.7)	*p* < 0.002
Apgar 15 min	9.9 (9.9–10.1)	9.9 (9.8–10.0)	10.0 (9.9–10.1)	ns
Maternal age (years)	25.4 (24.3–26.5)	26.0 (25.0–27.0)	26.8 (25.4–28.1)	ns
Peak bilirubin value, distribution of cases	<300 2	<300 1		
	300–339 5	300–339 3		
	340–399 29	340–399 32		
	400–449 14	400–449 23		
	>450 3	>450 3		
Gender m/f	35/22	39/32	30/40	ns

**Notes.**

CI Confidence Interval of the mean

No difference between the affected-HB and unaffected HB-groups was found in parity, the percentage of small-for-date, mother’s smoking habits during pregnancy, maternal diabetes, the number of X-ray investigations during pregnancy, past miscarriages, maternal blood pressure, mother’s weight gain during pregnancy, glycosuria, toxemia, mother’s marital status or the hereditary traits (diabetes, neurological, sensory deficits). Among all cases with HB, 54 received phototherapy and 79 had blood exchange transfusions (in six cases more than one transfusion). The number of treatments was equal in the affected-HB group (46 treated, 11 non-treated) and the unaffected-HB HB group (55 treated, 16 non-treated). Neither were there any significant differences in the start time, duration of HB or the peak bilirubin value. ABO blood group incompatibility was present in 40 cases and Rh incompatibility in one. The treated and non-treated HB subjects were equal in terms of cognitive testing, school performance, and other assessments at 9 years of age indicating that treatment decisions did not explain the measured outcome differences.

### Medical events and development at birth, and 9 years of life

There were no differences in the children’s hospitalizations, traumas, or other somatic complaints and diseases in follow-up. The body-mass-index BMI of the child was similar in the three groups at age 9 years (see Table 2). The groups did not differ in the amount of reported past developmental problems within the family (44% in the affected-HB, 28% in the unaffected-HB and 34% in the controls).

**Table 2:** Comparison of cognitive functioning, health and family situation in the affected and unaffected groups with hyperbilirubinemia (HB) as well as the healthy controls at 9 years of age. The significance for differences between the three groups in univariate comparisons is also given.

	Affected-HB *n* = 57	Unaffected-HB *n* = 71	Controls *n* = 70	
	Mean (95% CI)	Mean (95% CI)	Mean (95% CI)	Univariate test across the three groups
**Cognitive functioning**				
WISC VIQ	104.8 (102.0–107.6)	119.2 (116.6–121.7)	120.0 (117.4–122.5)	*p* < 0.0001
WISC PIQ	109.1 (106.1–112.2)	118.2 (115.5–121.0)	122.4 (119.7–125.2)	*p* < 0.0001
WISC FSIQ	107.4 (104.6–110.3)	121.0 (118.4–123.5)	123.3 (120.8–125.9)	*p* < 0.0001
ITPA total	33.8 (33.0–34.7)	37.7 (36.9–38.4)	37.2 (36.5–38.0)	*p* < 0.0001
Reading test	8.3 (8.1–8.6)	8.8 (8.6–9.0)	8.8 (8.6–9.0)	*p* < 0.003
Spelling test	7.0 (6.8–7.3)	7.9 (7.6–8.2)	8.5 (8.3–8.8)	*p* < 0.0001
**Health and family situation**				
BMI	29.8 (28.5–31.1)	31.6 (30.5–32.7)	31.5 (30.4–32.6)	ns
Social distress score	4.3 (3.4–5.1)	2.7 (2.0–3.4)	3.1 (2.4–3.8)	*p* < 0.02
Socio-economic status	2.4 (2.1–2.7)	1.9 (1.6–2.1)	2.0 (1.8–2.3)	*p* < 0.04

**Notes.**

WISC Wechsler Intelligence Scale for Children VIQ Verbal Intelligence Quotient PIQ Performance Intelligence Quotient FSIQ Full Scale Intelligence Quotient ITPA Illinois Test of Psycholinguistic Abilities BMI Body Mass Index CI Confidence Interval of the mean

The family’s socio-economic status was the highest and the social distress scores the lowest in the unaffected HB group differentiating it from the affected HB group but not from the control group in pair-wise comparisons. The affected-HB group did not differ from the controls. The significance levels were low and considering the multiple tests performed, the findings are not considered meaningful.

The performance of the groups in the Illinois Test of Psycholinguistic Abilities (ITPA), the Reading and spelling tests as well as the Wechsler Intelligence Scale for Children (WISC) performed at the age of 9 years is presented in Table 2 demonstrating significant differences between the three groups (MANOVA Wilks’ lambda *p* < 0.0001). In pair-wise comparisons the affected-HB group differed from the controls in all tests conducted; the unaffected-HB group differed from the controls only in spelling test (*p* < 0.003). The frequency of remedial instruction in preschool and school until nine years of age had been 86% in affected-HB, 44% in unaffected-HB, and 28% in the control groups (*p* < 0.0001).

The results show that despite similar medical history and growth pattern, many children in the HB group managed less well in the psychological tests than the control group. This is in line with a previous cross-sectional study of this cohort (Michelsson, Donner & Lindahl, 1988). In that study, where cases under 2500 g and 35–36 weeks at birth were also included, half of the HB group presented with writing difficulties at the age of 9 years (Helenius, 1987). They also had more problems at school, had lower grades, and more often attended special classes (Michelsson, Donner & Lindahl, 1988). In the present study, where the pre-term cases were excluded, cognitive tests showed the same result. The mean IQs were above average in all groups but as the controls performed at a high average range, possibly due to somewhat outdated norms and the so-called Flynn effect (Flynn, 1987), the differences were significant. As expected based on the classification performed, the affected-HB children also needed more special education and help in school. Thus, the negative effect of HB to cognition was not caused by the borderline cases of low weight and gestational age.

In contrast to our observations, studies reporting childhood outcomes of HB have mostly found no significant consequences (Culley et al., 1970; Newman & Klebanoff, 2002; Ip et al., 2004; Gamaleldin et al., 2011). In our cohort 55% of the HB subjects belonged to the unaffected group and these subjects were comparable to controls in most variables studied, supporting the general view. However, a smaller percentage was affected, resembling the cohort in Denmark (Maimburg et al., 2010), emphasizing the need to identify separate subgroups. Conflicting findings may also depend on the different ages of children studied. An important factor influencing the clinical picture is the maturation of frontal cortices, which is not complete until after adolescence, perhaps in the ages between 15 and 20 years of age (Paus, 2005). The rate of this development is poorly known, and maturation rates may differ between genders, which should be considered when interpreting results of follow-up studies. The association of the cortical maturation with IQ scores is also unclear (Burgaleta et al., 2014). A third potential factor is the Hawthorne effect: very active treatment is likely to be given in centers where also research is done, which may skew results in the direction of less serious consequences.

### Academic and occupational achievement reported at 30 years of age

All but 2 subjects in all groups combined had graduated from first level education (the first obligatory nine years). However, 11% of the affected-HB subjects reported having required remedial teaching or a special class during those nine school years, in contrast to only one child in the control group and none in the unaffected-HB group (*p* < 0.004). The proportion of subjects who had received the diploma at the end of the secondary education (after 12 years of school) was 30% in the affected-HB group, 70% in the unaffected-HB and 75% in the control group (*p* < 0.0001). Within the HB group, the relative risk of not completing secondary education was more than two-fold (*RR* = 2.49, 95% confidence interval 1.65–3.73, *p* < 0.0001) for a person with affected-HB compared to an unaffected-HB.

**Figure 2:**
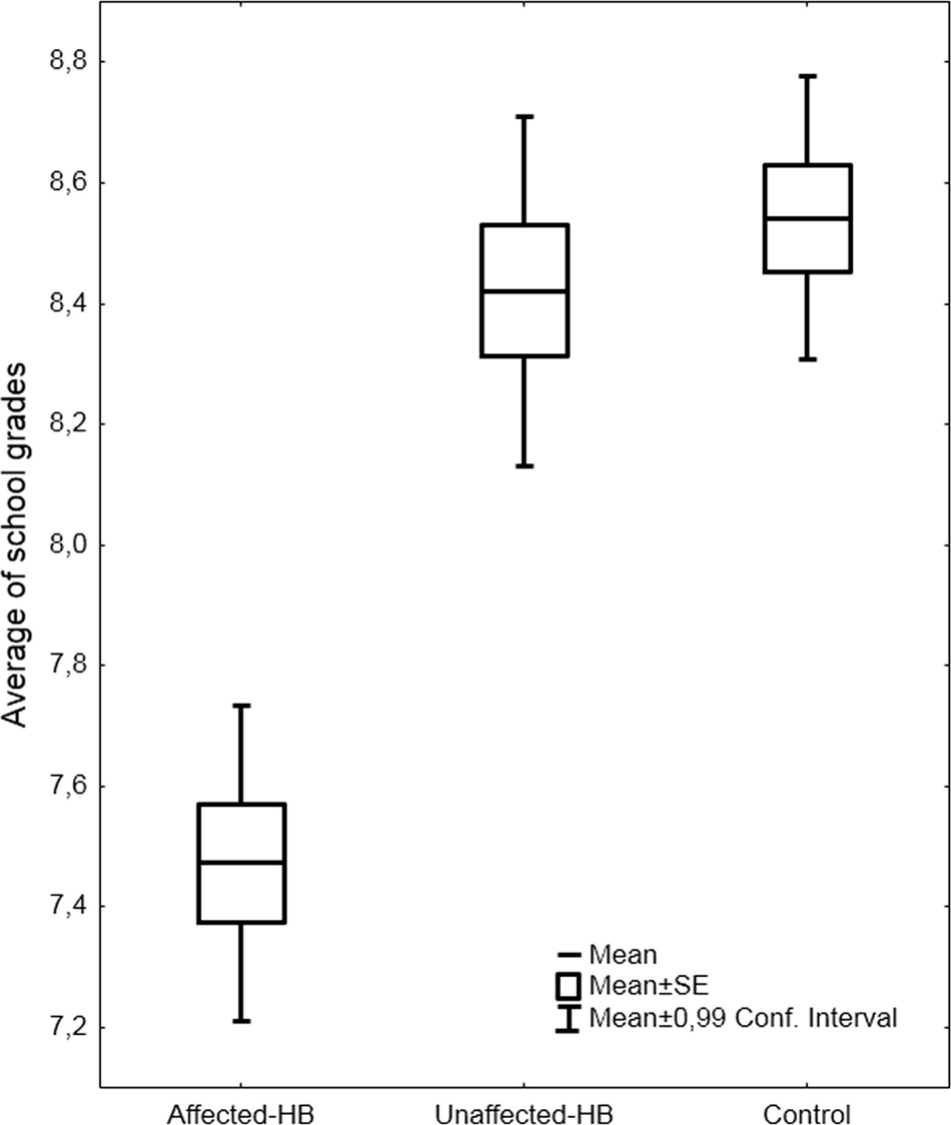
Average school grades at school graduation. The average school grades (mean, SEM, 99% confidence intervals) of subjects with hyperbilirubinemia and controls.

At the point of school graduation the mean of all school marks (the total average of all subjects) was significantly different in the three groups *F*(2,207) = 29.1, *p* < .0001, the affected-HB group having the lowest grades and differing from the other two groups in pair-wise comparison (*p* < .0001) (see Fig. 2).

The proportion of subjects who by the age of 30 had completed an academic degree was 11% in the affected-HB group, 32% in the unaffected-HB and 31% in the control group (*p* < 0.0001). When other forms of tertiary education (applied universities) were also included, the relative risk of not completing tertiary education was still elevated (*RR* = 1.57, 1.13–2.13, *p* < 0.004) for a person with affected-HB compared to an unaffected-HB.

At 30 years of age 86%, 93% and 90% of the groups (affected-HB, unaffected-HB and controls, respectively) were working or studying full time, showing a similar outcome. At the other end of the spectrum, however, 11% of the affected-HB, 3% of the controls and none of the unaffected-HB groups were unemployed (*p* < 0.006).

The results indicate that the subgroup of affected-HB had poorer academic achievement, i.e., lower mean school grades, and less potential to graduate from secondary or tertiary education. There was also a greater risk for later unemployment. Corresponding findings in academic and occupational failure are reported in other causes of high birth risk, e.g., low birth weight and asphyxia (Hack & Klein, 2006; Arpino et al., 2010; Stuart, Otterblad Olausson & Källen, 2011). The academic achievement is widely regarded as an objective and a sensitive measure, and it is influenced not only by general intelligence but also by memory, motivation, executive functions and social skills. A similar course of underachievement has been found, e.g., in adults with ADHD who may have significantly less education than expected based on their IQ, and lower occupational levels than expected based on their education (Biederman et al., 2008).

### Social functioning, health and substance use at 30 years of age

Marital status and number of offspring was equal in all groups. Still, social dissatisfaction score was higher (see Table 3) in the affected-HB group compared to the controls in pair-wise comparison (*p* < 0.002).

**Table 3:** Results from the questionnaire at 30 years for the affected and unaffected groups with hyperbilirubinemia (HB) as well as for the healthy controls. The significance for differences between the three groups in univariate comparisons is also given.

	Affected-HB *n* = 57	Unaffected-HB *n* = 71	Controls *n* = 70	
	Mean (95% CI)	Mean (95% CI)	Mean (95% CI)	Univariate test across the three groups
**Health and well-being**				
Social dissatisfaction score	3.0 (2.5–3.5)	2.3 (1.9–2.8)	1.8 (1.3–2.3)	*p* < 0.003
General health problems score	2.4 (2.1–2.8)	2.0 (1.7–2.3)	1.8 (1.5–2.2)	ns
Psychiatric problems score	0.2 (0.1–0.3)	0.08 (0.0–0.2)	0.06 (0.0–0.2)	ns
Alcohol consumption score	2.8 (2.0–3.5)	1.4 (0.7–2.1)	1.6 (0.9–2.3)	*p* < 0.03
**Behavioral functioning**				
Current inattention symptoms	2.78 (1.97–3.59)	2.56 (1.88–3.24)	1.91 (1.22–2.60)	ns
Current hyperactive–impulsive symptoms	2.96 (2.12–3.80)	2.86 (2.16–3.56)	2.36 (1.65–3.07)	ns
Childhood inattention symptoms	5.52 (4.34–6.70)	2.79 (1.79–3.78)	2.16 (1.15–3.17)	*p* < 0.0001
Childhood hyperactive–impulsive symptoms	4.40 (3.26–5.53)	2.68 (1.72–3.63)	2.25 (1.28–3.21)	*p* < 0.02
**Cognitive functioning**				
Subjective persisting problems present absent	13 (23%) 43 (77%)	7 (10%) 64 (90%)	5 (7%) 64 (93%)	*p* < 0.03

**Notes.**

CI Confidence Interval of the mean

The general health problem score was similar in the three groups and there were no differences in the reported incidences of traumas, fractures and concussions.

Alcohol consumption was more uncontrolled in the affected-HB group compared to the controls in pair-wise comparison. Particularly, past episodes of driving while intoxicated were reported by 20% of the affected-HB group but only by 6% and 9% in the unaffected-HB and control groups respectively. The use of illicit drugs was similar in the three groups, 10% of all reported having used at any time in the past.

The results indicate higher alcohol consumption in the affected-HB group. Similar tendencies in association with developmental deficits have also been observed previously (Biederman et al., 2008). Excessive alcohol consumption may be a significant confounding factor in analyzing vocational outcome. The affected-HB group had the highest average in a score measuring alcohol use but the number of subjects reporting consumption at a level that could be classified as abuse however was small. There were only a few current users of illicit drugs at the time the questionnaire was completed and the frequency of ever using any substance was also low. Incidence of substance abuse in Finland has been among the lowest in Europe for decades, although a rise has occurred in the last 10 years (Varjonen et al., 2012). We conclude that alcohol and substance abuse does not explain the differences observed in vocational outcome.

### Cognitive and psychiatric status at 30 years of age

Ongoing cognitive problems, all symptoms combined, were more frequent in the affected-HB group compared to the two other groups (Table 3). Sixteen per cent of the affected-HB group reported reading difficulty, 9% writing, 9% speech and 9% mathematical difficulty, and smaller percentages other learning or perceptual problems. In the subjective ADHD symptom rating scales (see Table 3) the three groups differed significantly (MANOVA Wilks’ lambda, *p* < 0.004). The affected-HB group scored higher for childhood inattention compared to both the unaffected-HB and the control groups (*p* < 0.001), and also higher for childhood hyperactivity/impulsivity compared to the controls (*p* < 0.01). The unaffected-HB group scored higher in childhood inattention compared to the controls (*p* < 0.002). The difference was not significant for the current symptoms rating scales.

No differences in number of self-reported psychiatric problems between the groups were found. The use of sedatives or hypnotics was reportedly similar in all groups.

The results indicate that the various learning disabilities found in a subgroup of the HB cases in childhood continue into adulthood. In our study, 23% of the affected-HB subjects reported persisting cognitive complaints at the age of 30 years, reading difficulties being reported most frequently. This is in line with the previous results at 9 years of age (Helenius, 1987). In addition to learning disabilities, the subjects reported subjective ADHD symptoms more frequently than the controls. In the retrospective childhood ADHD symptom rating scale both HB groups (affected and unaffected) reported higher scores of inattention than controls, but the difference was no longer significant in adulthood. The childhood hyperactive/impulsive symptoms were higher in the affected-HB group, but they too levelled off by adulthood, resembling the typical pattern for ADHD symptoms (Biederman, Mick & Faraone, 2000; Wilens, Biederman & Spencer, 2002). ADHD is suggested to be more common among subjects with pre- and perinatal risk factors (Strang-Karlsson et al., 2008; Gustafsson & Källén, 2011; Halmøy et al., 2012) although contradictory findings have also been reported (Silva, Colvin & Hagemann, 2013). Similarly mixed evidence has emerged regarding the link between ADHD and hyperbilirubinemia (Jangaard et al., 2008; Kuzniewicz, Escobar & Newman, 2009). In our study, the ADHD diagnosis cannot reliably be made based on questionnaire data, but ADHD symptoms appeared to be a part of the disability spectrum.

## Conclusions

Our aim was to study long term consequences of neonatal hyperbilirubinemia at 30 year-old adults followed up from birth. To exclude the confounding effects of preterm birth, we included only newborns ≥ 2500 g birth weight and ≥ 37 weeks of gestation. The subjects with neonatal hyperbilirubinemia were divided into subgroups based on the presence (affected HB) or absence (unaffected HB) of diagnosed cognitive or behavioral disabilities in childhood, and compared with healthy controls. Our results indicate that neonatal HB has negative consequences in adult age. Nearly half of the HB group were affected by cognitive abnormalities in childhood and continued to experience problems in adulthood. This was apparent in academic achievement and the ability to complete secondary and tertiary education. Also, the subgroup of affected HB reported persisting cognitive complaints, e.g., problems with reading, writing and calculus. Childhood symptoms of hyperactivity/impulsivity and inattention were more common in HB groups, but in adulthood the symptom levels were equal. The affected HB still perceived lower levels of subjective wellbeing as well as social adaptability, and reported more poorly controlled drinking; findings that may reflect a more profound tendency of poor adjustment.

The strength of our study is that our control and HB groups were remarkably similar at birth in terms of medical data and absence of risk factors, other than HB. Our base cohort’s limit for inclusion in the low birth weight group was 2000 g and inclusion to any group did not have a fixed limit of weeks of gestation. Consequently, there were a few neonates both in the control and the HB groups, whose gestational age was 35–36 weeks and/or birthweight between 2000 and 2500 g. In order to avoid any further bias we also excluded these borderline cases. The growth environment of the children was also very similar in the groups, as the parents’ social status, education, size of family, type of housing were comparable at birth and at 9 years of age. Further, the children were born in the same hospital, lived in the same metropolitan area, and attended the same schools. We also tested for attritional bias, and found none. Therefore we think that confounding factors are well controlled without need for statistical modeling. Our results should be well generalizable in otherwise uncomplicated HB cases, even in the era of more modern therapeutic possibilities.

A limitation in our study is that a questionnaire is a rather insensitive tool that has sampling and data processing challenges (Gilbody, House & Sheldon, 2001; Olsen et al., 2001; Jaddoe et al., 2006; Jaddoe et al., 2010). However, mailed inquiries are widely used and allow statistical comparison of parallel groups. Furthermore, in our study the cases had also been clinically carefully examined from birth up to age 9 years, which increases the reliability of our data. The main outcomes reported here, i.e., need for remedial teaching, low school grades and academic underachievement, are objective and we regard the differences between our groups credible. Still, although statistically significant differences were found, the results are indicative rather than proof of specific types of cognitive consequences due to the low specificity of a questionnaire.

The symptoms we found, e.g., decreased social adjustment, symptoms of the ADHD spectrum, and the ability to achieve goals when in an instructive environment (primary education with special tuition) but not when independent planning and execution of tasks are required (higher education or occupational environment) bear resemblance to impairment of the fronto-striatal system. The pathogenesis of encephalopathy by unconjugated bilirubin is poorly understood but it seems to involve oxidative stress, a disruption of the blood–brain barrier and the consequent damage of neuronal cells (Palmela et al., 2011). Brain pathology typically following bilirubin neurotoxicity has been suggested to involve the basal ganglia, particularly globus pallidus, as well as subthalamic nuclei, hippocampus, diencephalon, midbrain, pontine and brain stem nuclei and cerebellum (Johnson & Bhutani, 2011; Koziol, Budding & Chidekel, 2013), locations which take part in a wide variety of motor, sensory and cognitive functions. Fronto-subcortical circuits (Cummings, 1993) connect the frontal cortex to the basal ganglia and these circuits participate in executive functions (attention control, response inhibition, planning and execution). In this respect, HB has common elements with the processes that are active in other perinatal disorders that cause brain damage, e.g., ischemia-asphyxia and immaturity and/or low birth weight (e.g., Volpe, 2005; Volpe, 2012). Clearly, speculations on frontal dysfunction in HB warrant further studies with thorough neuropsychological testing and neuroimaging.

We conclude that neonatal HB is a recognizable cause of adult age neurobehavioral problems, severe enough to have negative occupational and quality of life effects.

## References

[ref-1] American Psychiatric Association (1994). Diagnostic and statistical manual of mental disorders, fourth edition: DSM-IV.

[ref-2] Arpino C, Compagnone E, Montanaro M, Cacciatore D, De Luca A, Cerulli A, Di Girolamo S, Curatolo P (2010). Preterm birth and neurodevelopmental outcome: a review. Child’s Nervous System.

[ref-3] Barkley RA, Murphy KR (1998). Attention-deficit hyperactivity disorder: a clinical workbook.

[ref-4] Bax M, Whitmore K (1973). Neurodevelopmental screening in the school-entrant medical examination. Lancet.

[ref-5] Biederman J, Mick E, Faraone SV (2000). Age-dependent decline of symptoms of attention deficit hyperactivity disorder: impact of remission definition and symptom type. American Journal of Psychiatry.

[ref-6] Biederman J, Petty CR, Fried R, Kaiser R, Dolan CR, Schoenfeld S, Doyle AE, Seidman LJ, Faraone SV (2008). Educational and occupational underattainment in adults with attention-deficit/hyperactivity disorder: a controlled study. The Journal of Clinical Psychiatry.

[ref-7] Burgaleta M, Johnson W, Waber DP, Colom R, Karama S (2014). Cognitive ability changes and dynamics of cortical thickness development in healthy children and adolescents. NeuroImage.

[ref-8] Culley P, Powell J, Waterhouse J, Wood B (1970). Sequelae of neonatal jaundice. British Medical Journal.

[ref-9] Cummings J (1993). Frontal-subcortical circuits and human behavior. Archives of Neurology.

[ref-10] Dubowitz LMS, Leibowitz D, Goldberg CA (1977). Clinical screening test for assessment of intellectual development in four and five-year-old children. Developmental Medicine & Child Neurology.

[ref-11] Ebbesen F, Ehrenstein V, Traeger M, Nielsen GL (2010). Neonatal non-hemolytic hyperbilirubinemia: a prevalence study of adult neuropsychiatric disability and cognitive function in 463 male Danish conscripts. Archives of Disease in Childhood.

[ref-12] Flynn JR (1987). Massive IQ gains in 14 nations: what IQ tests really measure. Psychological Bulletin.

[ref-13] Frostig M, Lefever W, Whittlesey JRB (1966). Administration and scoring manual for the marianne frostig test of visual peception.

[ref-14] Gamaleldin R, Iskander I, Seoud I, Aboraya H, Aravkin A, Sampson PD, Wennberg RP (2011). Risk factors for neurotoxicity in newborns with severe neonatal hyperbilirubinemia. Pediatrics.

[ref-15] Gilbody SM, House AO, Sheldon T (2001). Routinely administered questionnaires for depression and anxiety: systematic review. BMJ.

[ref-16] Goodenough F (1926). Measurement of intelligence by drawings.

[ref-17] Gustafsson P, Källén K (2011). Perinatal, maternal, and fetal characteristics of children diagnosed with attention-deficit-hyperactivity disorder: results from a population-based study utilizing the Swedish Medical Birth Register. Developmental Medicine and Child Neurology.

[ref-18] Hack M, Klein N (2006). Young adult attainments of preterm infants. JAMA.

[ref-19] Halmøy A, Klungsøyr K, Skjærven R, Haavik J (2012). Pre- and perinatal risk factors in adults with attention-deficit/hyperactivity disorder. Biological Psychiatry.

[ref-20] Helenius M (1987). Writing disabilities and cognitive functions at the age of nine years in a neonatal high-risk group. Early Child Development and Care.

[ref-21] Hokkanen L, Launes J, Michelsson K (2013). The Perinatal Adverse events and Special Trends in Cognitive Trajectory (PLASTICITY)—pre-protocol for a prospective longitudinal follow-up cohort study. F1000Research.

[ref-22] Ip S, Chung M, Kulig J, Brien RO, Sege R, Glicken S, Maisels MJ, Lau J (2004). American Academy of Pediatrics Subcommittee on Hyperbilirubinemia (2004). An evidence-based review of important issues concerning neonatal hyperbilirubinemia. Pediatrics.

[ref-23] Jaddoe VWV, Mackenbach JP, Moll HA, Steegers EAP, Tiemeier H, Verhulst FC, Witteman JC, Hofman A (2006). The generation R study: design and cohort profile. European Journal of Epidemiology.

[ref-24] Jaddoe VWV, van Duijn CM, van der Heijden AJ, Mackenbach JP, Moll HA, Steegers EAP, Tiemeier H, Uitterlinden AG, Verhulst FC, Hofman A (2010). The generation R study: design and cohort update 2010. European Journal of Epidemiology.

[ref-25] Jangaard KA, Fell DB, Dodds L, Allen AC (2008). Outcomes in a population of healthy term and near-term infants with serum bilirubin levels of > or = 325 micromol/L (> or = 19 mg/dL) who were born in Nova Scotia, Canada, between 1994 and 2000. Pediatrics.

[ref-26] Johnson L, Bhutani VK (2011). The clinical syndrome of bilirubin-induced neurologic dysfunction. Seminars in Perinatology.

[ref-27] Kaplan M, Bromiker R, Hammerman C (2011). Severe neonatal hyperbilirubinemia and kernicterus: are these still problems in the third millennium?. Neonatology.

[ref-28] Kirk SA, McCarthy J, Kirk WD (1968). The Illinois test of psycholinguistic abilities.

[ref-29] Koziol LF, Budding DE, Chidekel D (2013). Hyperbilirubinemia: subcortical mechanisms of cognitive and behavioral dysfunction. Pediatric Neurology.

[ref-30] Kuzniewicz M, Escobar GJ, Newman TB (2009). No association between hyperbilirubinemia and attention-deficit disorder. Pediatrics.

[ref-31] Kuzniewicz M, Newman TB (2009). Interaction of hemolysis and hyperbilirubinemia on neurodevelopmental outcomes in the collaborative perinatal project. Pediatrics.

[ref-32] Lindahl E, Michelsson K (1986). Neurodevelopmental significance of minor and major congenital anomalies in neonatal high risk children. Neuropediatrics.

[ref-33] Lindahl E, Michelsson K (1987). Prognosis of neonatal “at risk” infants at early school-age: a comprehensive outcome score as a measure of impairment. Early Child Development and Care.

[ref-34] Lindahl E, Michelsson K, Donner M (1988). Prediction of early school-age problems by a preschool neurodevelopmental examination of children at risk neonatally. Developmental Medicine and Child Neurology.

[ref-35] Maimburg RD, Bech BH, Vaeth M, Møller-Madsen B, Olsen J (2010). Neonatal jaundice, autism, and other disorders of psychological development. Pediatrics.

[ref-36] Michelsson K, Donner M, Lindahl E (1988). Neurodevelopmental screening of 5-year-old children. European Journal of Pediatrics.

[ref-37] Michelsson K, Lindahl E (1984). Nine-year follow-up of infants weighing 1500 g or less at birth. Acta Paediatrica Scandinavica.

[ref-38] Michelsson K, Lindahl E (1987). School failure in a group of nine-year-old children who neonatally belonged to a high risk group. Early Child Development and Care.

[ref-39] Michelsson K, Lindahl E, Kalverboer AF, Hopkins B, Geuze R (1993). Relationship between perinatal risk factors and motor development at the ages of 5 and 9 years. Motor development in early and later childhood.

[ref-40] Michelsson K, Lindahl E, Helenius M, Parre M (1988). Five and nine year check up of 314 children with neonatal hyperbilirubinaemia. Early Child Development and Care.

[ref-41] Michelsson K, Noronen M (1983). Neurological, psychological and articulatory impairment in five-year-old children with a birthweight of 2000 g or less. European Journal of Pediatrics.

[ref-42] Michelsson K, Ylinen A, Donner M (1981). Neurodevelopmental screening at five years of children who were at risk neonatally. Developmental Medicine and Child Neurology.

[ref-43] Michelsson K, Ylinen A, Saarnivaara A, Donner M (1978). Occurrence of risk factors in newborn infants. A study of 22,359 consecutive cases. Annals of Clinical Research.

[ref-44] Newman TB, Klebanoff M (2002). 33 272 infants, 7-year follow-up: total serum bilirubin, transfusions reexamined. Pediatrics.

[ref-45] Nilsen ST, Finne PH, Bergsjø P, Stamnes O (1984). Males with neonatal hyperbilirubinemia examined at 18 years of age. Acta Paediatrica Scandinavica.

[ref-46] Olsen J, Melbye M, Olsen SF, Sørensen TI, Aaby P, Andersen AM, Taxbøl D, Hansen KD, Juhl M, Schow TB, Sørensen HT, Andresen J, Mortensen EL, Olesen AW, Søndergaard C (2001). The Danish National Birth Cohort—its background, structure and aim. Scandinavian Journal of Public Health.

[ref-47] Palmela I, Cardoso FL, Bernas M, Correia L, Vaz AR, Silva RFM, Fernandes A, Kim KS, Brites D, Brito MA (2011). Elevated levels of bilirubin and long-term exposure impair human brain microvascular endothelial cell integrity. Current Neurovascular Research.

[ref-48] Paus T (2005). Mapping brain maturation and cognitive development during adolescence. Trends in Cognitive Sciences.

[ref-49] Polanczyk G, de Lima M, Horta B, Biederman J, Rohde L (2007). The worldwide prevalence of ADHD: a systematic review and metaregression analysis. American Journal of Psychiatry.

[ref-50] Ruoppila I, Roman K, Vasti M (1968).

[ref-51] Ruoppila I, Roman K, Vasti M (1969).

[ref-52] Seidman DS, Paz I, Stevenson DK, Laor A, Danon YL, Gale R (1991). Neonatal hyperbilirubinemia and physical and cognitive performance at 17 years of age. Pediatrics.

[ref-53] Sgro M, Campbell D, Shah V (2006). Incidence and causes of severe neonatal hyperbilirubinemia in Canada. Canadian Medical Association Journal.

[ref-54] Shapiro SM (2003). Bilirubin toxicity in the developing nervous system. Pediatric Neurology.

[ref-55] Shaywitz SE, Shaywitz BA (2005). Dyslexia (specific reading disability). Biological Psychiatry.

[ref-56] Silva D, Colvin L, Hagemann E, C Bower (2013). Environmental risk factors by gender associated with attention-deficit/hyperactivity disorder. Pediatrics.

[ref-57] StatSoft, Inc. (2013). http://www.statsoft.com.

[ref-58] Stott DH, Moyes FA, Henderson SA (1972). A test of motor impairment.

[ref-59] Strang-Karlsson S, Räikkönen K, Pesonen A-K, Kajantie E, Paavonen EJ, Lahti J, Hovi P, Heinonen K, Järvenpää AL, Eriksson JG, Andersson S (2008). Very low birth weight and behavioral symptoms of attention deficit hyperactivity disorder in young adulthood: the Helsinki study of very-low-birth-weight adults. The American Journal of Psychiatry.

[ref-60] Stuart A, Otterblad Olausson P, Källen K (2011). Apgar scores at 5 minutes after birth in relation to school performance at 16 years of age. Obstetrics and Gynecology.

[ref-61] Varjonen V, Tanhua H, Forsell M, Perälä R (2012).

[ref-62] Volpe JJ (2005). Encephalopathy of prematurity includes neuronal abnormalities. Pediatrics.

[ref-63] Volpe JJ (2012). Neonatal encephalopathy: an inadequate term for hypoxic-ischemic encephalopathy. Annals of Neurology.

[ref-64] Wechsler D (1949). Wechsler intelligence scale for children.

[ref-65] Wilens TE, Biederman J, Spencer TJ (2002). Attention deficit/hyperactivity disorder across the lifespan. Annual Review of Medicine.

[ref-66] Willcutt EG, Betjemann RS, McGrath LM, Chhabildas NA, Olson RK, DeFries JC, Pennington BF (2010). Etiology and neuropsychology of comorbidity between RD and ADHD: the case for multiple-deficit models. Cortex.

